# Supplementary material for: TIGIT blockade improves anti-Mycobacterium tuberculosis immunity
Source: PLoS Pathog. 2025 Jun 17;21(6):e1013228. doi: 10.1371/journal.ppat.1013228 (PMC12173411; doi:10.1371/journal.ppat.1013228)
Supplement: S1 Table — (DOCX) [file ppat.1013228.s003.docx]

Supplementary material

**S1 Table. Demographic and clinical features of enrolled participants**

| **Characteristic** | **ATB** | **LTBI** | **HC** |
| --- | --- | --- | --- |
|  | (*n* = 57) | (*n* = 15) | (*n* = 25) |
| Male, n (%) | 34 (60.0%) | 7 (46.7%) | 7 (28.0%) |
| Age (years), median (IQR) | 46 (29) | 52 (19) | 31 (20)^a,b^ |
| BCG vaccinated, n (%) | 50 (87.7%) | 14 (93.3%) | 24 (96.0%) |
| IGRA-positive, n (%) | 51 (89.5%) | 15 (100%) | 0 |
| Definite diagnosis^#^, n (%) | 35 (61.4%) | NA | NA |
| No anti-tuberculosis treatment used on enrollment, n (%) | 35 (61.4%) | NA | NA |
| Comorbidity, n (%) |  |  |  |
| Cardiovascular diseases | 1 (1.75%) | 1 (6.67%) | 0 |
| Diabetes | 4 (7.02%) | 0 | 0 |
| Chronic hepatitis | 0 | 1 (6.67%) | 0 |

^#^, A definite diagnosis of tuberculosis is based on a positive result of pathological examinations, including smear acid-fast staining, culture and nucleic acid-based detection of *Mycobacterium tuberculosis*. ^a^, a statistically significant difference between ATB and HC; ^b^, a statistically significant difference between LTBI and HC. ATB, active pulmonary tuberculosis; LTBI, latent tuberculosis infection; HC, healthy control; BCG, bacillus Calmette-Guerin; IQR, interquartile range; NA, not applicable.
