# Supplementary material for: TIGIT blockade improves anti-Mycobacterium tuberculosis immunity
Source: PLoS Pathog. 2025 Jun 17;21(6):e1013228. doi: 10.1371/journal.ppat.1013228 (PMC12173411; doi:10.1371/journal.ppat.1013228)
Supplement: S3 Table — (DOCX) [file ppat.1013228.s005.docx]

Supplementary material

**S3 Table.** **Amino acid sequence of** **synthetic overlapping peptides of ESAT-6, CFP-10 and RD2 proteins.**

| Antigen protein | Peptide | Position | Amino acid sequence^a^ | Length(AA) |
| --- | --- | --- | --- | --- |
| ESAT6 | ESAT6-P1 | P1-20 | MTEQQWNFAGIEAAASAIQG | 20 |
|  | ESAT6-P2 | P12-31 | EAAASAIQGNVTSIHSLLDE | 20 |
|  | ESAT6-P3 | P23-42 | TSIHSLLDEGKQSLTKLAAA | 20 |
|  | ESAT6-P4 | P34-53 | QSLTKLAAAWGGSGSEAYQG | 20 |
|  | ESAT6-P5 | P45-64 | GSGSEAYQGVQQKWDATATE | 20 |
|  | ESAT6-P6 | P56-75 | QKWDATATELNNALQNLART | 20 |
|  | ESAT6-P7 | P67-86 | NALQNLARTISEAGQAMAST | 20 |
|  | ESAT6-P8 | P78-95 | EAGQAMASTEGNVTGMFA | 18 |
| CFP10 | CFP10-P1 | P1-20 | MAEMKTDAATLAQEAGNFER | 20 |
|  | CFP10-P2 | P12-31 | AQEAGNFERISGDLKTQIDQ | 20 |
|  | CFP10-P3 | P23-42 | GDLKTQIDQVESTAGSLQGQ | 20 |
|  | CFP10-P4 | P34-53 | STAGSLQGQWRGAAGTAAQA | 20 |
|  | CFP10-P5 | P45-64 | GAAGTAAQAAVVRFQEAANK | 20 |
|  | CFP10-P6 | P56-75 | VRFQEAANKQKQELDEISTN | 20 |
|  | CFP10-P7 | P67-86 | QELDEISTNIRQAGVQYSRA | 20 |
|  | CFP10-P8 | P78-100 | QAGVQYSRADEEQQQALSSQMGF | 23 |
| RD2 proteins |  |  |  |  |
| Rv1986 | Rv1986-P9 | P91-110 | AWRPVALIPSGATPVRLAEV | 20 |
|  | Rv1986-P15 | P157-176 | WFATLGFGAGRLRGLFTNPG | 20 |
|  | Rv1986-P16 | P168-187 | LRGLFTNPGSWRILDGLIAV | 20 |
| Rv1988 | Rv1988-P4 | P34-53 | ELVFDIGAGEGALTAHLVRA | 20 |
|  | Rv1988-P11 | P111-130 | TLLAPNSGLVAADLVLQRAL | 20 |
| Rv1983 | Rv1983-P4 | P290-309 | PTSTILVDTGSAGLVVSPED | 20 |

^a^ Amino acid sequences are shown in single letter code from the N terminus to the C terminus.
