# Supplementary figures and images for: TIGIT blockade improves anti-Mycobacterium tuberculosis immunity
Source: PLoS Pathog. 2025 Jun 17;21(6):e1013228. doi: 10.1371/journal.ppat.1013228 (PMC12173411; doi:10.1371/journal.ppat.1013228)

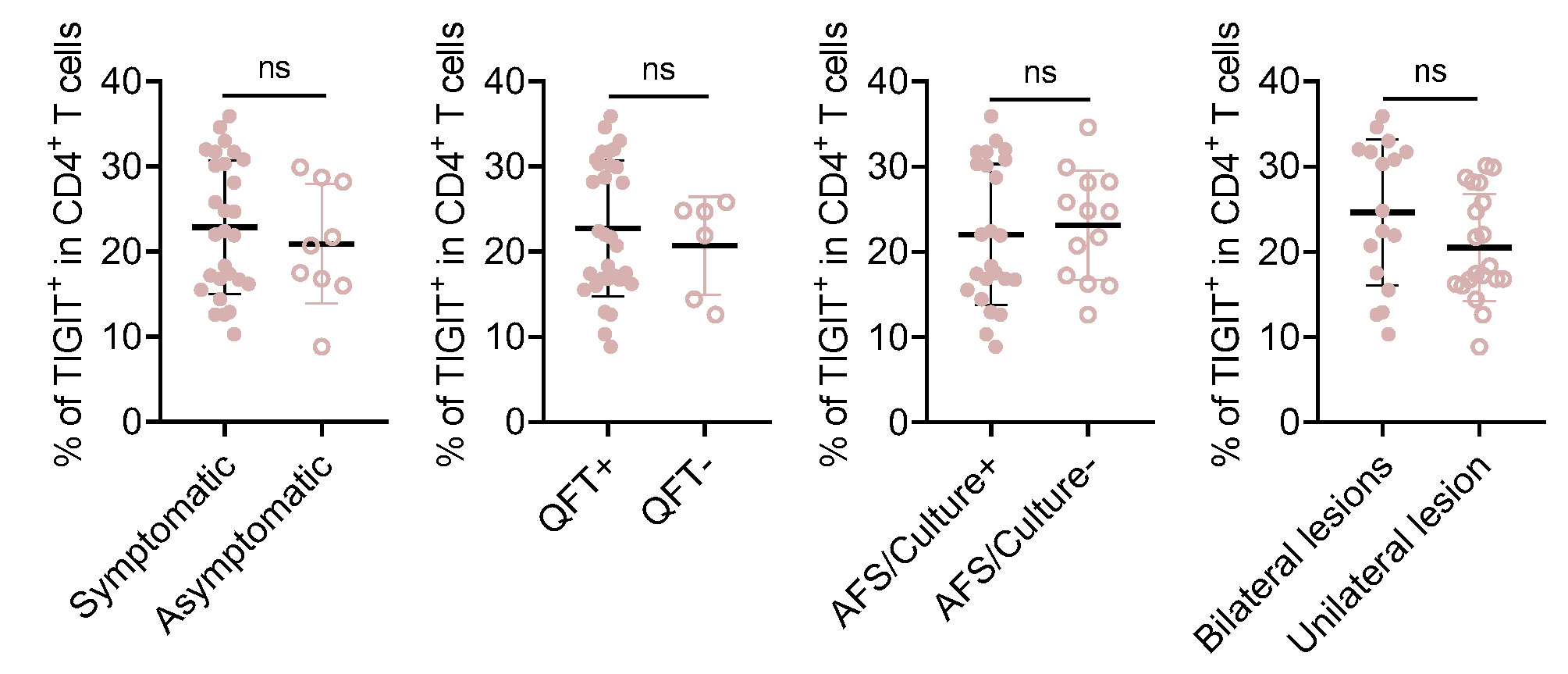

Supplement: S1 Fig — Symptomatic refers to cough over 2weeks, chest pain, fatigue, weight loss, fever, night sweats, dyspnea, and/or hemoptysis. Data are presented as median with interquartile range. Statistical significance (P < 0.05) was obtained using a Mann-Whitney U test. ns, not statistically significant. (TIF) [file ppat.1013228.s006.tif]

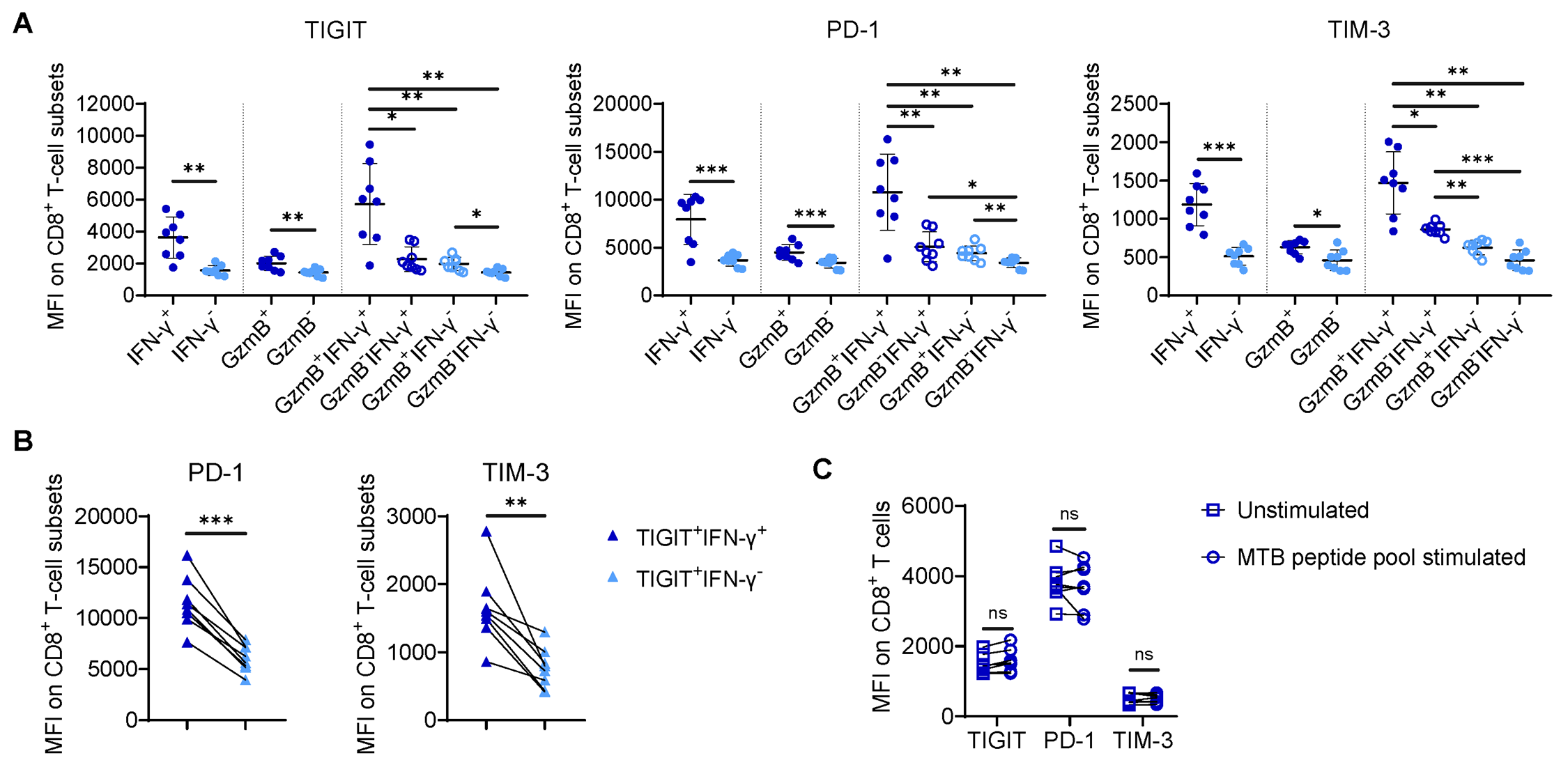

Supplement: S2 Fig — Expression of TIGIT and other inhibitory receptors on MTB-responsive CD8+ T cells. (A) Expression of TIGIT, PD-1 and TIM-3 on IFN-γ- and/or granzyme B-producing CD8+ T cells, and (B) PD-1 and TIM-3 expression on IFN-γ+ and IFN-γ− TIGIT+ CD8+ T-cell subsets from individuals with ATB (n = 8) upon MTB peptide pool stimulation. (C) Comparison of TIGIT, PD-1 and TIM-3 expression on CD8+ T cells from individuals with ATB (n = 8) with or without MTB peptide pool stimulation. Data are presented as mean ± standard deviation in panel A, and as individual values in panel B and C. Statistical significance (P < 0.05) was obtained using a one-way ANOVA with Bonferroni post-test or paired t test. *, P < 0.05; **, P < 0.01; ***, P < 0.001; ns, not statistically significant. (TIF) [file ppat.1013228.s007.tif]

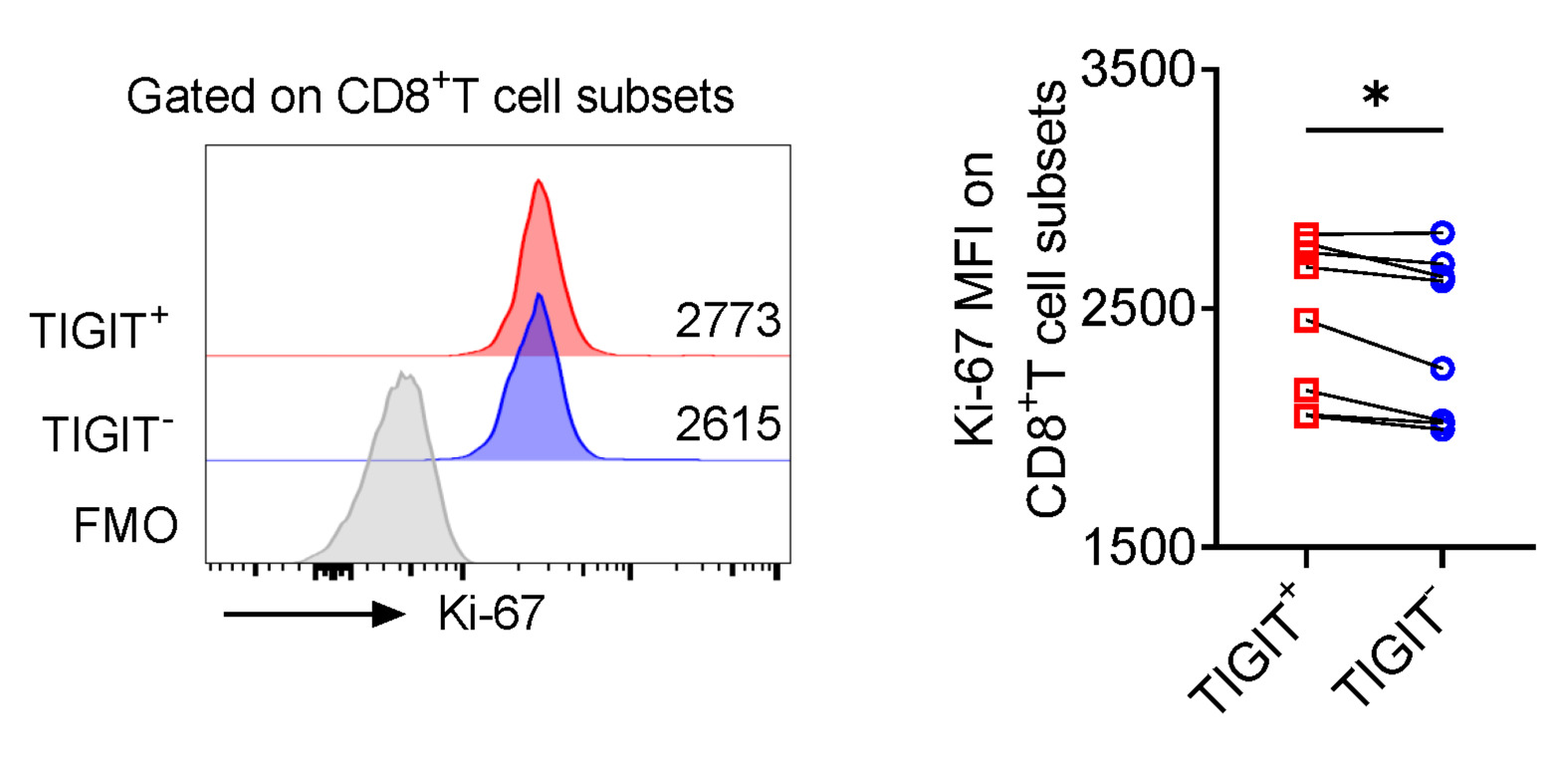

Supplement: S3 Fig — Data are presented as individual values. Statistical significance (P < 0.05) was obtained using a Wilcoxon signed rank test. *, P < 0.05. (TIF) [file ppat.1013228.s008.tif]

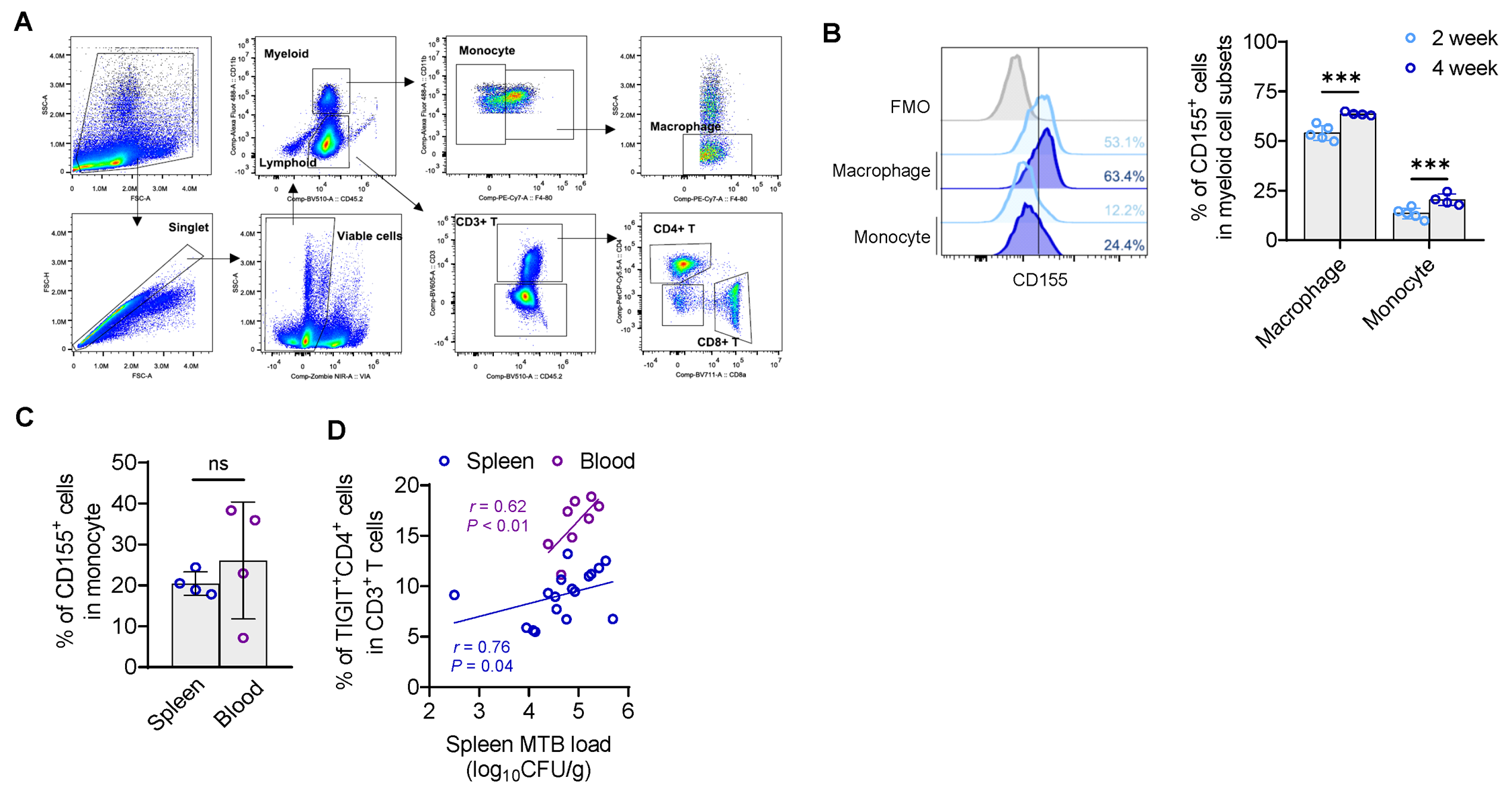

Supplement: S4 Fig — (A) Gating strategy and (B) representative histograms and cumulative frequencies of CD155+ cells among spleen macrophages and monocytes in isotype control antibody treated mice during MTB infection for 2 weeks (n = 5) and 4 weeks (n = 4). (C) Cumulative frequencies of CD155+ cells among monocytes in spleen (n = 5) and blood (n = 4) samples from MTB infected mice at 4 weeks. (D) Scatter plots of the frequencies of TIGIT+ CD4+ cells in spleen (n = 17) and peripheral (n = 8) T cells against spleen MTB viable counts in infected mice. Data are presented as mean ± standard deviation in panels B and C, and individual values in panel D. Statistical significance (P < 0.05) was obtained using a Wilcoxon signed rank test or Spearman correlation analysis. ***, P < 0.001; ns, not statistically significant. (TIF) [file ppat.1013228.s009.tif]

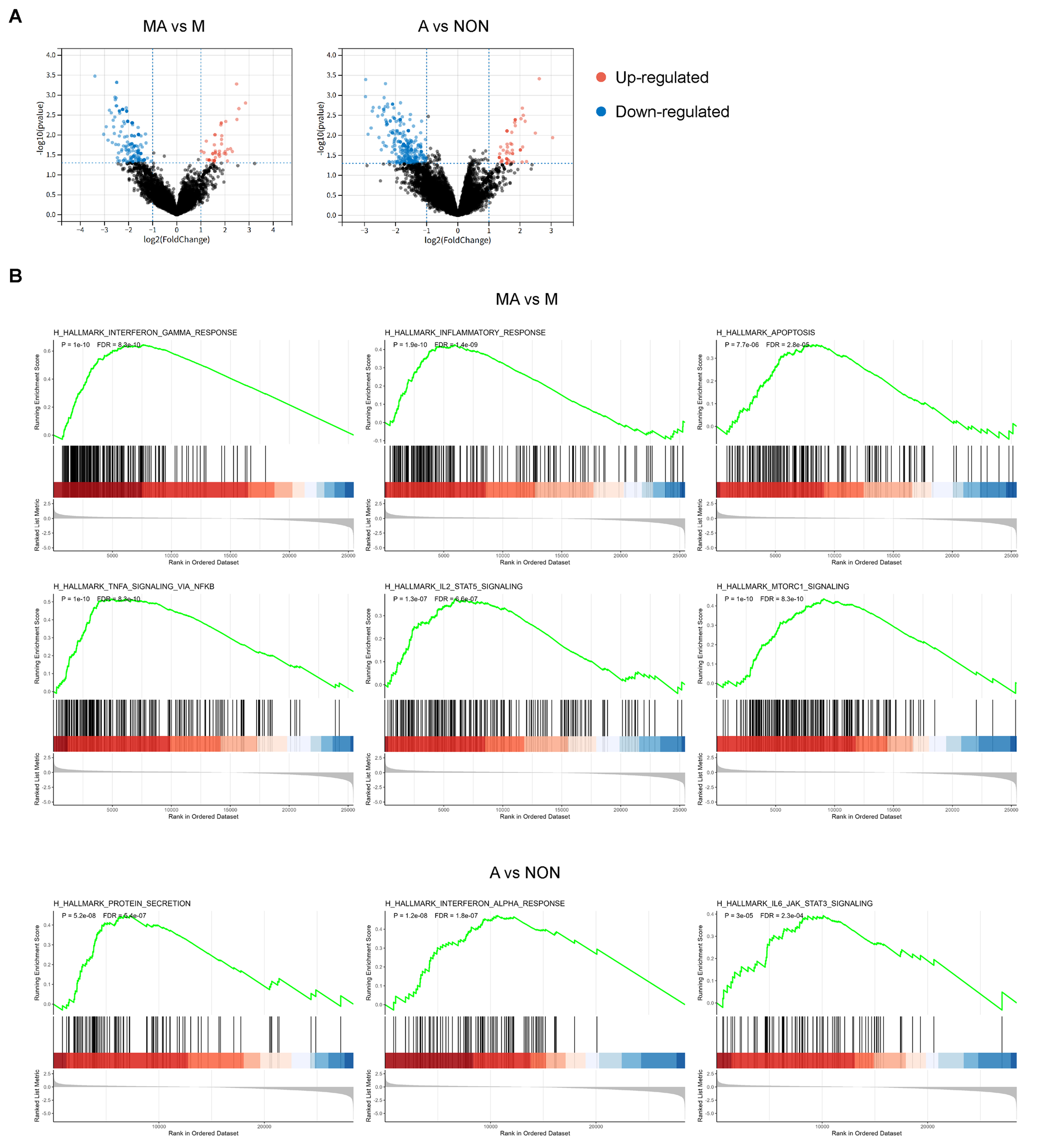

Supplement: S5 Fig — (A) Volcano plots of differentially expressed gene (DEGs) related to TIGIT blockade in CD8+ T cells with or without MTB peptide stimulation. (B) Gene sets enriched in CD8+ T cells in response to in vitro TIGIT blockade. MA, MTB peptide stimulation plus in vitro TIGIT blocking antibody treatment; M, MTB peptide stimulation alone; A, in vitro TIGIT blocking antibody treatment alone; NON, culture medium alone. (TIF) [file ppat.1013228.s010.tif]
