# Supplementary material for: TIGIT blockade improves anti-Mycobacterium tuberculosis immunity
Source: PLoS Pathog. 2025 Jun 17;21(6):e1013228. doi: 10.1371/journal.ppat.1013228 (PMC12173411; doi:10.1371/journal.ppat.1013228)
Supplement: Attachment — Submitted filename: 1Response to Reviewers 0426.docx [file ppat.1013228.s012.docx]

Prof. David M. Lewinsohn,

Academic Editor

Prof. Michael Wessels

Section Editor

Prof. Sumita Bhaduri-McIntosh

Editor-in-Chief

Prof. Michael Malim

Editor-in-Chief

PLOS Pathogens

April 24, 2025

Dear editors:

We appreciate the thoughtful and insightful reviews of our manuscript (submission ID: PPATHOGENS-D-24-02617 [EMID:96ac96f99f504f7e]). We have provided additional data and addressed each of the points raised the reviewers as indicated below. The changes we have made in the main text are shown in red.

Reviewers' Comments:

Part I - Summary

Reviewer #1: In this manuscript, Zhou and colleagues investigated the role of TIGIT in tuberculosis. The authors found higher TIGIT expression on circulating CD8 T cells in blood from patients with ATB than compared to patients with LTBI and healthy controls. The authors conclude that circulating TIGIT+ CD8+ T cells resemble an activated phenotype rather than an exhausted phenotype. The authors also assess the use of anti-TIGIT antibodies in vitro and in vivo. Incubation with anti-TIGIT antibody in vitro resulted in increased IFNg and granzyme B production, albeit small, in CD8 T cells. Treatment of H37Rv-infected mice anti-TIGIT antibody initiated one week after infection resulted in a reduction in lung CFU, but no reduction of spleen CFU. This study is the first to characterize TIGIT in blood and lung lesions from patients with TB. While these finding are novel and the in vivo anti-TIGIT antibody experiments are interesting, the discussion regarding the nuance of TIGIT over the course of infection (i.e., chronic and acute infection) as well as providing context of the literature of other immune checkpoint receptors (e.g., PD-1) in TB is under-developed.

We appreciate the favorable review of our manuscript and the helpful comments by this reviewer.

Reviewer #2: Summary: The authors characterize TIGIT expression on CD4 and CD8 T cells in patients with active and latent tuberculosis to assess their contribution to TB control. They use a series of assays to evaluate immune cell phenotype, effector function, proliferative capacity and transcriptional profile to interrogate T cell subsets with respect to TIGIT expression. Additionally, they evaluated the correlation between TIGIT expression and clinical severity of TB in patients. They present convincing data that TIGIT blockade improves proliferation and cytokine/cytoxic function in CD8+ T cells in samples from patients with active TB. Although, TIGIT has been described as an exhaustion marker for T cells in the literature, the authors present data that TIGIT expression is a marker of activation in TB rather than representing a state of exhaustion. The authors further support their human data with in vivo studies in mice demonstrating that TIGIT blockade results in reduced Mtb CFU but does not induce more immunopathology in the lungs. Their study contributes to our understanding of the role of immune checkpoints in tuberculosis immunity and immunopathology. Overall this is an interesting study, well written and clear scientific reasoning. However, several key issues should be addressed:

We thank the reviewer for the positive comment of our manuscript and the constructive suggestions.

Part II – Major Issues: Key Experiments Required for Acceptance

Reviewer #1:

1) There is limited acknowledgment in the introduction and discussion to the nuance of immune checkpoint receptors as it relates to immune activation, exhaustion and tuberculosis. For example, immune checkpoint receptors like PD-1 are upregulated during acute infection during initial immune activation. In contrast, in chronic infections, co-expression of multiple immune checkpoint receptors is used to identify exhausted cells. It would improve context to provide discussion of the literature of TIGIT over the course of infection and in contrast to other immune checkpoint receptors. In addition, it is unclear in the manuscript whether TIGIT is framed as an activation marker or an exhaustion marker. At times in the manuscript, TIGIT is compared to PD-1, which would imply its relation to immune exhaustion, and yet there is limited discussion regarding the body of work investigating PD-1 in TB (PMID: 33452107-, 20624978-, 32091388-, 30651320-, 29891540).

We apologize for the lack of discussion in the main text.

We have revised the corresponding part in the “Introduction” and “Discussion” section to discuss more about the nuance of immune checkpoint receptors and current knowledge about TIGIT (Line 96-98, 279-287).

On basis of phenotypic, transcriptional and TIGIT blockade assay results, we consider TIGIT as an activation marker with regulatory effect in ATB. Correlated statement has been added to the “Discussion” section (Line 339-341).

Discussion about current knowledge in the role of PD-1 in tuberculosis and the mentioned references has been added to the corresponding part in the “Discussion” section (Line 341-354).

2) In Fig 1D, TIGIT expression is compared between symptomatic and asymptomatic patients with TB. Active TB is typically defined as exhibiting symptoms, so it is not possible to be asymptomatic with active TB. In addition, symptoms are not defined for “symptomatic” patients. In Fig 1E, “other pulmonary infection” is not defined and, thus, the rationale for making such a comparison is not clear.

Thanks for the insightful comment. All patients with ATB enrolled in this study are proven cases with either a positive pathological or histological test result of tuberculosis, or clinical diagnosed cases with typical chest radiological evidence, supplemented by clinical symptoms of pulmonary tuberculosis, or a positive tuberculin skin test or interferon-γ release assay result, in accordance with WHO consolidated guidelines on tuberculosis and our lasted national diagnostic criteria of pulmonary tuberculosis (WS 288-2017). The presence of clinical symptoms is not a requirement for diagnosis of tuberculosis, as the diagnostic accuracy of symptoms is unfavorable, and people are more and more aware that individuals with pulmonary tuberculosis can be asymptomatic (1).

The definition of symptomatic ATB patients is ATB patients presented with cough over 2 weeks, chest pain, fatigue, weight loss, fever, night sweats, dyspnea, and/or hemoptysis. Considering the impact of host immune response on the clinical presentation and outcomes of MTB infection, we speculate there might be difference in the T-cell immune between symptomatic and asymptomatic ATB patients. Therefore, we compared TIGIT expression between these subgroups. We have revised the corresponding part in the figure legends of Figure 1 and Figure S1. If the reviewer still opposes the grouping based on symptom, we agree to remove the related result from Figure 1D.

In Figure 1E, individuals with other pulmonary infection comprised two cases with pulmonary cryptococcosis and one case with actinomyces pulmonary infection. These patients were included based on chest CT manifestation of solitary nodules in unilateral or bilateral lobes before surgery and chronic granulomatous lesions in lung surgical excision biopsy. We have revised the corresponding part in the “Materials and Methods” section (Line 398-401).

3) There is limited rationale provided in the introduction as to why the authors chose to focus their study on CD8 T cells.

We apologize for the lack of explanation in the main text. Our choice to focus on CD8^+^ T cells was based on the differential expression of TIGIT observed in CD8^+^ T cells using PBMC from individuals with ATB or LTBI, or without MTB infection, as demonstrated in the first part of the “Results” section. We have also added current understanding and research gaps in the study of CD8^+^ T-cell immune response in MTB infection to the “introduction” section (Line 81-91).

Reviewer #2:

1. The human data evaluates TIGIT on bulk CD4 and CD8 T cell responses but does not show data on the role of TIGIT in antigen specific CD8+ T cell function, which is important for interpreting their ability to have a protective impact on TB immunity. Additionally, among effector molecule-expressing T cells, what frequency are TIGIT+ or TIGIT-? This question is slightly different than what the authors an answer in Figure 2. This will help to answer what is the role of TIGIT expressing T cells in TB since it is well established that antigen-specific T cells are important for TB control. Additionally, how does peptide stimulation alter the TIGIT, TIM-3, PD-1 profiles? What are the TIGIT, TIM-3, PD-1 profiles of antigen specific cells (Figure 2).

We appreciate the insightful comment. The wide diversity of HLA haplotypes in the enrolled patients is highly likely to result in HLA-mismatching between these donors and the HLA-restricting alleles used to generate the tetramers, which has been proven limits the consistency between epitope-pool ICS and tetramer-pool assays (2). Therefore, we adopted the approach of functional response detection upon peptide pool stimulation as previous studies (3-6). Cytokine-producing cells are considered antigen-specific cells and subject to further functional or phenotypic assessment of MTB-specific CD8^+^ T cells.

To evaluate the role of TIGIT in MTB-specific CD8^+^ T cells, we compared the proportion of effector molecule-expressing CD8^+^ T cells upon MTB peptide pool stimulation between *in vitro* TIGIT blockade and isotype control groups. As showed in Figure 4A, we observed higher levels of IFN-γ- and granzyme B-producing cells after TIGIT blockade, which suggested a negative regulatory role of TIGIT on MTB-specific CD8^+^ T cells.

We analyzed the expression of TIGIT on effector molecule-expressing CD8^+^ T cells upon MTB peptide pool stimulation. Results showed that TIGIT is expressed at a significantly higher level on either IFN-γ- or granzyme B-producing CD8^+^ T-cell subset, and the multifunctional subset had the highest TIGIT expression (Figure S2A). It further supports our observation that TIGIT is more likely a marker of activation and TIGIT-expressing CD8^+^ T cells from patients with ATB exhibited a polarized activated effector phenotype.

Analysis of TIM-3 and PD-1 revealed a similarly high expression of these checkpoint receptors on MTB-responsive CD8^+^ T cells (Figure S2A), as well as on the TIGIT^+^ IFN-γ^+^ CD8^+^ T-cell subset compared to the TIGIT^+^ IFN-γ^−^ subset (Figure S2B). However, we did not observe any significant alteration in the expression level of TIGIT, PD-1 or TIM-3 on CD8^+^ T cells from ATB patients after short-term 24h stimulation (Figure S2C), suggesting the upregulation of these checkpoint receptors likely requires continuous TCR stimulation with a longer duration or a combination of other stimulus, such as cytokines.

Data are shown in Figure S2, and we have revised the corresponding part in the “Results” section (Line 159-163).

2. Line 178/ Figure 4 (line 692): The title of section/figure is misleading because the co-culture was performed with BCG and not Mtb. Although there is some overlap between BCG and Mtb, BCG lacks many of the pathogenic factors that contribute the Mtb replication and immune suppression. Therefore, the authors need to use Mtb as this may generate different data than what is presented. Additionally, the CFU differences between isotype and TIGIT blockade are very modest (Figure 4C). More image contrast is needed in the representative images (Figure 4C) to observe/assess the visual differences.

We appreciate the helpful suggestion. We have revised the title of the section and figure, and have enhanced contrast in the representative images in Figure 4C to better show the visual differences.

Co-culture assay using MTB is required to be performed in the BSL-3 laboratory in accordance with our biosafety regulations. However, due to constraints in our BSL-3 laboratory facilities, neither fluorescence-activated nor magnetic-activated cell sorting could be performed currently. In substitution, we therefore chose to use the attenuated *Mycobacterium bovis* BCG, which preserved higher virulence than the avirulent H37Ra strain. Our data could not show the exact effect of *in vitro* TIGIT blockade on killing of intracellular infected MTB, but it still supported an enhanced ability in clearance of *Mycobacterium* by CD8^+^ T cells. We will try to conduct this additional analysis in future study once the condition permits.

3. Figure 5: similar to the human data, there is no assessment of antigen specific T cells responses that could be evaluated with Mtb peptide stimulation in the murine cells. Additionally, there is no effector function readout (IFN-gamma, TNFa, granzyme, etc.). Although there is data on T cells in the blood and spleen, T cell lung responses are integral in the control of Mtb and are not provided. Functional assays should be performed on infected mouse lung homogenates to elucidate why TIGIT blockade results in a CFU difference in the lungs but not the spleen.

We appreciate the helpful suggestion. Due to constraints in our BSL-3 laboratory facilities, we were unable to perform lymphocytes isolation, stimulation and ICS using infected mouse lungs at that time. Based on available resources and current experimental conditions, we have selected an alternative approach, and assessed the effect of *in vivo* TIGIT blockade on the immune response in lungs by performing quantitative PCR analysis using cryopreserved lung homogenates. The transcription levels of *Ifng*, *Tnf*, *Gzmb*, and *Prf1* were compared between TIGIT blockade group and isotype control group. Notably, the TIGIT blockade group demonstrated significantly elevated *Ifng* transcript levels compared to the control group, which helped explain the reduced MTB load in mouse lungs, and was consistent with our finding in CD8^+^ T cell function upon *in vitro* TIGIT blockade. We admit that current analysis could not elucidate the exact effect of *in vivo* TIGIT blockade on CD8^+^ T cells, but it still revealed the change in immune response in the lungs. We will try to conduct these additional analysis of MTB-specific T cells in mouse lung in future study once the condition permits.

Data are shown in Figure 5F, and we have revised the corresponding part in the “Result”, “Materials and Methods”, and “Figures and figure legends” sections (Line 243-246, 418-419, 547-560).

4. Figure 5D/ Supplementary Table 2/Animal studies methods: the anti-mouse TIGIT blocking antibody details are not provided (clone, manufacturer), also in vivo dosing and timing of 7 administrations should be explicitly stated.

We apologize for the lack of description in the main text. We have added the information of anti-mouse antibodies for *in vivo* treatment to Table S2, and stated the dosing and strategy of *in vivo* antibody treatment in the corresponding part of the “Materials and Methods” section (Line 410-414).

5. Figures 5B and 5C: The axis should be labeled “% of TIGIT+ in T cell subsets” not “in CD8+ T cell subsets”, since this is a comparison between bulk CD3+ T cells and CD4+ and CD8+ T subsets.

We apologize for the mistakes in the figure. We have corrected the axis title in Figure 5B and 5C accordingly.

6. Clarify content of the MTB peptide pool (peptide sequence/ protein origin). Are these overlapping peptides? To which antigens do these peptides correspond? This information is needed to inform readers about expected stimulation patterns especially among TB researchers.

Thank you for your kind reminder. We have added the protein origin and amino acid sequences of the MTB peptide pool used in this study to supplementary S3 Table, and the corresponding part in the “Materials and Methods” section (Line 445-447).

7. Figure 3: Why was bulk RNAseq performed based on co-expression of TIGIT and PD-1 in ATB patients only? Flow data suggested that PD-1 had minor impact on TIGIT+ cells. RNAseq would have been more informative if populations from Mtb-peptide stimulated, and TIGIT blockade groups were compared to non-stimulated conditions. Alternatively, sequencing could have been performed between ATB, LTBI, and HC TIGIT+ CD8+ T cell populations. This would have been more insightful to make claims regarding TIGIT role in the context of Mtb.

We appreciate the question. We observed a significantly higher proportion of PD-1-expressing cells within the TIGIT^+^CD8^+^ T cell subset, as shown in Figure 2C, which suggested co-expression of PD-1 with TIGIT on CD8^+^ T cells from ATB patients. PD-1 has been reported to exert a negative regulatory effect on T-cell function (7-9). To control this confounding factor in the analysis of RNA-seq, we sorted CD8^+^ T cells based on co-expression of TIGIT and PD-1, and analyzed DEGs from comparison between TIGIT^+^PD-1^+^and TIGIT^−^PD-1^+^ and comparison between TIGIT^+^PD-1^−^ and TIGIT^−^PD-1^−^ CD8^+^ T cell subsets. We have revised the corresponding part in the “Result” section (Line 171-175).

We also appreciate the helpful suggestion. Accordingly, we cultured PBMC from ATB patients in the presence of MTB peptide pool plus TIGIT blocking antibody (referred as MA group), MTB peptide pool alone (M group), TIGIT blocking antibody alone (A group), or culture medium alone (NON group) for 24h. Then, CD8^+^ T cells were isolated by magnetic-activated cell sorting. Total RNA extracted from sorted CD8^+^ T cells were converted to cDNA libraries and sequenced. Transcriptional analysis showed that TIGIT blockade led to a total of 293 and 365 DEGs in MTB-peptide stimulated (MA vs M) and unstimulated (A vs NON) CD8^+^ T cells (Figure S5A). GSEA analysis was then performed to explore the potential impact of TIGIT on CD8^+^ T-cell immune response. Results showed that gene sets associated with biological process, including protein secretion and response to IFN-α, and IL-6-JAK-STAT3 signaling were significantly enriched in CD8^+^ T cells after blocking TIGIT (Figure S5B). Additionally, in CD8^+^ T cells stimulated with MTB peptides, TIGIT blockade could further induce expression of genes associated with response to IFN-γ, inflammatory response and apoptosis, and downstream pathway, including NF-κB signaling, IL-2-STAT5 signaling and mTORC1 signaling (Figure S5B). Activation of T cells has been found to suppress tumor-infiltrating CD8^+^ T-cell response via inhibition of MAPK/ERK, NF-κB, and AKT/mTOR signaling pathway (10, 11). These results indicated the possible mechanisms of the regulatory effect of TIGIT on CD8^+^ T cells, especially on MTB-specific CD8^+^ T cells.

Data are shown in Figure S5, and we have revised the corresponding part in the “Results” and “Materials and Methods” sections (Line 211-220, 529-530, 543).

Part III – Minor Issues: Editorial and Data Presentation Modifications

Reviewer #1: 4) Mycobacterium tuberculosis is a Gram Positive Bacteria, not Gram Negative. The manuscript Section/Category should be changed to reflect this.

We appreciate the helpful suggestion, and we will change the manuscript Section/Category.

5) Refs 24 & 25 appear to cite dissertation work and not published or accepted manuscripts. Suggest removing these reference and related text.

We thank the kind reminder, and these references have been removed.

6) Line 80, insert “non-human” before primate.

We appreciate the helpful suggestion. The text has been revised accordingly.

7) Line 101, awkward wording: suggest changing “distinct MTB infection statuses” to “either ATB or LTBI”.

We appreciate the helpful suggestion. The text has been revised accordingly.

8) Line 126, insert “active” between “during” and “tuberculosis”.

We appreciate the helpful suggestion. The text has been revised accordingly.

9) Lines 188 & 449, change “homologous” to “autologous”.

We appreciate the helpful suggestion. The text has been revised accordingly.

10) Line 189, include the numeric difference in CFU between TIGIT blockade and isotype in the text.

We appreciate the helpful suggestion. A reduction of 0.61 to 0.85×10^4^/ml in BCG CFU counts in co-culture cell lysates of TIGIT blockade group has been stated in the text.

11) Line 197, change “MTB clearance” to “mycobacterial clearance”. Experimentally, TIGIT blockade reduced BCG CFU, which is Mycobacterium bovis not MTB.

We appreciate the helpful suggestion. The text has been revised accordingly.

12) Lines 200-201, change “the spleens” to “spleen”.

We appreciate the helpful suggestion. The text has been revised accordingly.

13) Line 202, change “CD4+ spleen T cells” to “splenic CD4+ T cells”.

We appreciate the helpful suggestion. The text has been revised accordingly.

14) Lines 209-210, change “with the MTB viable counts in infected mouse spleens” to “with viable splenic CFU”.

We appreciate the helpful suggestion. The text has been revised accordingly.

15) Line 213, include the numeric difference in CFU between TIGIT blockade and isotype in the text.

We appreciate the helpful suggestion. In comparison to isotype control, TIGIT blockade treatment reduced lung MTB load by 1.45±0.19 log_10_CFU per gram, while treatment with anti-PD-1 antibody increased lung MTB CFU counts by 0.41±0.17 log_10_CFU. We have revised the corresponding part.

16) Line 232, change “organ” to “tissue”

We appreciate the helpful suggestion. The text has been revised accordingly.

17) Line 242, typo – change to “Both”

We appreciate the helpful suggestion. The text has been revised accordingly.

18) Line 269, typo – change to “speculation”

We appreciate the helpful suggestion. The text has been revised accordingly.

We appreciate the insightful and helpful comments by the reviewers and have revised our manuscript accordingly. We believe that our manuscript has been improved significantly by these changes and hope you now find it suitable for publication in *PLOS Pathogens.*

Sincerely,

*Qiaoling Ruan*

Mailing address: 12 M. Wulumuqi Road Shanghai 200040, China

E-mail address: qlruan07@fudan.edu.cn

Phone: (+86) 21-52888123

Fax: (+86) 21-52888714

*Lingyun Shao*

Mailing address: 12 M. Wulumuqi Road Shanghai 200040, China

E-mail address: lingyun26@fudan.edu.cn

Phone: (+86) 13916900780

Fax: (+86) 21-52888714

**Reference**

1.	Trajman A, Campbell JR, Kunor T, Ruslami R, Amanullah F, Behr MA, et al. Tuberculosis. Lancet. 2025;405(10481):850-66.

2.	Lancioni C, Swarbrick GM, Park B, Nyendak M, Nsereko M, Mayanja-Kizza H, et al. Recognition of CD8(+) T-cell epitopes to identify adults with pulmonary tuberculosis. Eur Respir J. 2019;53(5).

3.	Day CL, Abrahams DA, Lerumo L, Janse van Rensburg E, Stone L, O'Rie T, et al. Functional capacity of Mycobacterium tuberculosis-specific T cell responses in humans is associated with mycobacterial load. J Immunol. 2011;187(5):2222-32.

4.	Day CL, Moshi ND, Abrahams DA, van Rooyen M, O'Rie T, de Kock M, et al. Patients with tuberculosis disease have Mycobacterium tuberculosis-specific CD8 T cells with a pro-apoptotic phenotype and impaired proliferative capacity, which is not restored following treatment. PLoS One. 2014;9(4):e94949.

5.	Pollock KM, Whitworth HS, Montamat-Sicotte DJ, Grass L, Cooke GS, Kapembwa MS, et al. T-cell immunophenotyping distinguishes active from latent tuberculosis. J Infect Dis. 2013;208(6):952-68.

6.	Rozot V, Patrizia A, Vigano S, Mazza-Stalder J, Idrizi E, Day CL, et al. Combined use of Mycobacterium tuberculosis-specific CD4 and CD8 T-cell responses is a powerful diagnostic tool of active tuberculosis. Clin Infect Dis. 2015;60(3):432-7.

7.	Kauffman KD, Sakai S, Lora NE, Namasivayam S, Baker PJ, Kamenyeva O, et al. PD-1 blockade exacerbates Mycobacterium tuberculosis infection in rhesus macaques. Sci Immunol. 2021;6(55).

8.	Shen L, Gao Y, Liu Y, Zhang B, Liu Q, Wu J, et al. PD-1/PD-L pathway inhibits M.tb-specific CD4(+) T-cell functions and phagocytosis of macrophages in active tuberculosis. Sci Rep. 2016;6:38362.

9.	Li J, Jin C, Wu C, Huang J. PD-1 modulating Mycobacterium tuberculosis-specific polarized effector memory T cells response in tuberculosis pleurisy. J Leukoc Biol. 2019;106(3):733-47.

10.	He W, Zhang H, Han F, Chen X, Lin R, Wang W, et al. CD155T/TIGIT Signaling Regulates CD8(+) T-cell Metabolism and Promotes Tumor Progression in Human Gastric Cancer. Cancer Res. 2017;77(22):6375-88.

11.	Li S, Ding J, Wang Y, Wang X, Lv L. CD155/TIGIT signaling regulates the effector function of tumor-infiltrating CD8+ T cell by NF-κB pathway in colorectal cancer. J Gastroenterol Hepatol. 2022;37(1):154-63.
